# Intra-articular injection of miRNA-1 agomir, a novel chemically modified miRNA agonists alleviates osteoarthritis (OA) progression by downregulating Indian hedgehog in rats

**DOI:** 10.1038/s41598-024-56200-4

**Published:** 2024-04-06

**Authors:** Pengcui Li, Yangyang Gao, Raorao Zhou, Xianda Che, Hang Wang, Lingling Cong, Pinpin Jiang, Dan Liang, Penghua Li, Chunfang Wang, Wenjin Li, Shengbo Sang, Qianqian Duan, Xiaochun Wei

**Affiliations:** 1 Department of Orthopaedic Surgery, The Second Hospital of Shanxi Medical University, Taiyuan, 030001 Shanxi China; 2 Department of Orthopaedic Surgery, Jincheng People’s Hospital, Jincheng, 048000 Shanxi China; 3 Shanxi Province Fenyang Hospital, Fenyang, 032200 Shanxi China; 4 Department of Experimental Animal Center, Shanxi Medical University, Taiyuan, 030001 Shanxi China; 5 Department of Stomatology, Shanxi Medical University, Taiyuan, 030001 Shanxi China; 6 Key Lab of Advanced Transducers and Intelligent Control System of the Ministry of Education and College of Information and Computer, Taiyuan University of Technology, Jinzhong, 030600 China

**Keywords:** MicroRNA-1, Intra-articular injection, Osteoarthritis, Indian hedgehog, Medical research, Molecular medicine

## Abstract

Our objective in this study is to determine whether intra-articular injection of miRNA-1 can attenuate the progression of OA in rats by down regulating Ihh. Knee chondrocytes were isolated from male Sprague–Dawley rats aged 2–3 days. Second-generation chondrocytes were transfected with miR-1 mimic and empty vector with lipo3000 for 6 h and then stimulated with 10 ng/mL IL-1β for 24 h. OA-related and cartilage matrix genes were quantified using real-time quantitative polymerase chain reaction (RT-qPCR). Two-month-old male Sprague–Dawley rats were divided into three groups (n = 30?): sham operation group + 50 µL saline, anterior cruciate ligament transection (ACLT) group + 50 µL miR-1 agomir (concentration), and control group ACLT + 50 µL miR-1 agomir. Treatment was started one week after the operation. All animals were euthanized eight weeks after the operation. X-rays and micro-CT were used to detect imaging changes in the knee joints. FMT was used to monitor joint inflammation in vivo. Safranin O staining was used to detect morphological changes in articular cartilage. Immunohistochemistry was used to detect Col2, Col10, metalloproteinase-13 (MMP-13). RT-qPCR was used to detect gene changes includingmiR-1, Col2, Col10, MMP-13, Ihh, Smo, Gli1, Gli2, and Gli3. Overexpression of miR-1 in IL-1β-stimulated chondrocytes reduced the levels of Ihh, MMP-13, and Col10 but increased the levels of Col2 and aggrecan. Intra-articular injection of miR-1 agomir reduced osteophyte formation, inflammation, and prevented cartilage damage. RT-qPCR results indicated that the miR-1 agomir increased articular cartilage anabolism and inhibited cartilage catabonism. miR-1 can attenuate the progression of OA by downregulating Ihh.

## Introduction

Osteoarthritis (OA) is the most common joint disease affecting 28% of population over the age of 60 globally^1^. OA seriously impair quality of life and often lead to disability^2,3^. The steady increase in the prevalence of OA globally is mainly due to the increase in per capita age and obesity^4^. OA will create a huge economic burden on individuals and society and will be a major challenge for public health in the future^5^. Traditionally, OA treatment consists of pain management with a joint replacement for end-stage disease^6–8^. This approach does not address morbidity related to early illness or restrictions of surgery. Therefore, early treatment of OA is necessary. Currently, there is no drug available to prevent OA development because we don’t understand the pathogenesis of OA completely.

Indian hedgehog (Ihh) is a member of the hedgehog (Hh) family, which controls cartilager development by regulating the conservative target genes, patched (Ptc) and Gli^9,10^. Studies have shown that the conditional absence of Ihh in mouse chondrocytes can slow OA progression^11^. Blocking Ihh signaling may be a therapeutic approach to preventing and treating OA articular cartilage degeneration because Ihh is a key component involved in OA progression. However, there are currently no Ihh inhibitors that can be used in human beings, as most Hh signaling chemical inhibitors cause severe side effects^12–14^. Therefore, there is a need to develop an Ihh inhibitor that does not cause any side effects.

Recently, miRNAs in the cartilage have attracted increasing attention. miRNAs are a class of endogenous noncoding RNAs^10–16^ 18–24, nucleotides in length, that post-transcriptionally repress the expression of related protein-coding genes. miRNAs can recognize and bind to the 3′-untranslated region of mRNA through complementary base pairing, and guide the RNA-induced silencing complex to degrade the target mRNA or inhibit its translation^15–17^. Our previous study showed that miR-1 delayed the progression of osteoarthritis by inhibiting Ihh in transgenic mice^18^. Therefore, downregulation of the Ihh gene expression by intra-articular injection of miR-1 may be an effective way of delaying the progression of OA. miRNA-1 agomir are special chemically modified miRNA agonists that inhibit the expression of target gene mRNA by mimicking endogenous miRNA. miRNA agomir are highly stable and active in animals and are easily enriched in target cells through cell membranes and interstitial spaces. Si et al. demonstrated the reduction in the progression of OA and maintenance of homeostasis in rats by intra-articular injection of miR-140^19^. In this study, we used rat knee chondrocytes to validate the in vitro effects of miR-1. We then used an intra-articular injection of miR-1 to verify whether it can overexpress miR-1 in vivo and inhibit the expression of Ihh to delay the progression of OA, to provide new therapeutic methods and strategies for OA treatment.

## Materials and methods

### Chondrocyte culture

Knee chondrocytes were isolated from male Sprague–Dawley (SD) rats aged 2–3 days and cultured in Dulbecco’s modified Eagle’s medium (DMEM). After the culture of the second generation, cells were seeded in 6-well plates.

To investigate the effect of miR-1 in vitro, we transfected miR-1 mimic and empty vector with lipo3000 into chondrocytes for 6 h, stimulated cells with 10 ng/mL IL-1β for 24 h, and then collected cells for real-time quantitative polymerase chain reaction (RT-qPCR).

### Construction of MiR-1 agomir and MiR-1 mimics

miR-1 agomir and miR-1 mimics were manufactured by Guangzhou RIboBio Co., Ltd (Guangzhou, China). miRNA agomirs are chemically modified miRNA agonists that modulate the expression of target mRNAs by mimicking endogenous miRNAs. miRNA agomirs have higher stability and activity in animals than common miRNA mimics and are more likely to be enriched in target cells through cell membranes and interstitial spaces. miRNA mimics can simulate a high-level expression of mature miRNA in cells to enhance the regulation of endogenous miRNA for gain-of-function research^19^.

### Establishment of rat OA model and transfection of miR-1

The rat OA model (n = 12) was established by right anterior cruciate ligament transection (ACLT)^20^, and the rats (n = 12) were randomly divided into two groups: ACLT + miR-1 agomir (n = 6) and ACLT + miR-1 agomir control (n = 6). The rats in the sham group (n = 6) only had the nodular sac cut, not the anterior cruciate ligament. The intra-articular cartilageinjection were performed one week after the surgery^19^. The sham operation, experimental, and control groups were injected with 50 µL PBS, 50 µL miR-1 agomir, and 50 µL miR-1 antagomir, respectively. After 8 weeks of modeling, all animals were euthanized. The method of anesthesia in rats is intraperitoneal injection of 3% sodium pentobarbital solution (1 ml/kg), and the euthanasia method is excessive anesthesia.

### Radiography

When the rats were killed in the 8th week after modeling, a small-animal X-ray apparatus (Faxitron UltraFocus, Arizona, USA) was used to observe radiographic changes in the right knee of rats. After the rats were anesthetized, X-rays of the right knee joints in the anterior and lateral positions were taken. The exposure time and kV settings were set at “full AUTO”. MicroCT scans (CT80; ScancoMedical AG, Brüttisellen, Switzerland) were performed with an integration time of 300 ms, a source voltage of 70 kV, and an isotropic nominal resolution of 10 μm. The volume of interest included the femoral condyles and epiphyseal bone as the upper limit and the tibial plateau and epiphyseal bone as the lower limit (approximately 12–15 mm).

### Histology

The right knee joints (three in each group) were taken, fixed with 4% paraformaldehyde for 48 h, and then decalcified in 10% EDTA for one and a half months. Each decalcified specimen was evenly divided along the coronal position and embedded in paraffin blocks. The cutting thickness was 5 µm. Then, ten 5-µm sections were taken at every 200 µm, and two sections were stained with safranin O/fast green at every interval. Three independent and blinded observers scored each slice using the International Osteoarthritis Research Society (OARSI) grading system and averaged the scores of the proximal tibia slices of each rat. The score ranged from 0 to 24, with higher scores indicating more severe degradation^21^.

### Immunohistochemistry

Col2, Col10, and metalloproteinase-13 (MMP-13) were identified using immunohistochemistry. After sections were dewaxed with xylene and hydrated with graded ethanol. An endogenous peroxidase blocker was used to block peroxidase activity for 10 min. 0.1% trypsin was used for 30 min to expose the antigenic determinants. Col2, Col10, or MMP-13 antibodies were incubated overnight at 4 °C. The sections were then incubated with antibodies conjugated to horseradish peroxidase (HRP) for 30 min at 37 °C. Images were captured using a Leica DM6B microscope (Leica).

### RT-qPCR

The cartilage of the tibia plateau and femoral condyle was scraped with a scalpel and ground with liquid nitrogen. The TRIzol reagent was used for total RNA extraction. The obtained RNA was then reverse transcribed into complementary DNA (cDNA) using PrimeScript™ RT Master Mix (Takara Bio, Inc.), and a portion of the samples was reverse transcribed into microRNA using the HyperScript™ III miRNA 1st strand cDNA Synthesis Kit (by stem-loop) (NovaBio, Shanghai, China). The cDNA samples were then subjected to qPCR amplification using the TB Green™ Premix Ex Taq™ II Kit (Takara), 2 × S6 miRNA SYBR qPCR mixture (NovaBio), and Biosystems™ QuantStudio™ 6 Flex Real-Time PCR System (Applied Biosystems; Thermo Fisher Scientific, Inc.). rRNA 18 s and U6 were used as internal controls for the mRNA and miRNA, respectively. The stem-loop primers for miR-1 were purchased from Qiagen. PrimeScript™ RT Master Mix was used to reverse transcribe the isolated RNA into complementary DNA (cDNA), and the TB Green™ Premix Ex Taq™ II and Applied Biosystems™ QuantStudio™ 6 Flex Real-Time PCR system were used for RT-qPCR. The reaction conditions for RT-qPCR amplification were 40 cycles at 95 °C for 30 s, 95 °C for 5 s, and 60 °C for 30 s, and the corresponding dissociation conditions were 95 °C for 15 s, 60 °C for 60 s, and 95 °C for 15 s. The primer sequences used are listed in Table 1.

**Table 1:** Primer sequences.

Species	Gene	DNA sequence
Rat	Col2	5ʹ-GGAGCAGCAAGAGCAAGGAGAAG-3ʹ
		5ʹ-GGAGCCCTCAGTGGACAGTAGAC-3ʹ
	Col10	5ʹ-GGATGCCTCTTGTCAGTGCTAACC-3ʹ
		5ʹ-TCATAGTGCTGCTGCCTGTTGTAC-3ʹ
	MMP-13	5ʹ-ATACGAGCATCCATCCCGAGACC-3ʹ
		5ʹ-AACCGCAGCACTGAGCCTTTTC-3ʹ
	Ihh	5ʹ-CTGCTGCTGCTGCTGCTTCTG-3ʹ
		5ʹ-GTAGGCAGGAGGCACGAGTTTACG-3ʹ
	Gli1	5ʹ-CGTTTGAAGGCTGTCGGAAGTCC-3ʹ
		5ʹ-GGTCACTGGCGTTGCTGAAGG-3ʹ
	Gli2	5ʹ-ACCTCCATCACCGTGCCTACC-3ʹ
		5ʹ-AGTCTTGACCTTGCTCCGCTTATG-3ʹ
	Gli3	5ʹ-ACAGCAACACCAGCACCATCAG-3ʹ
		5ʹ-CTTCAGCCTGCGATGCCTCAC-3ʹ
	Smo	5ʹ-TGGCTGACTGGCGGAACTCC-3ʹ
		5ʹ-CCAATGCTGCCCACAAAGAAACAC-3ʹ
	MiR-1	5ʹ-GCGCGTGGAATGTAAAGAAGT-3ʹ
		5ʹ-AGTGCAGGGTCCGAGGTATT-3ʹ
	U6	5ʹ-GCTCGCTTCGGCAGCACATATAC-3ʹ
		5ʹ-AGTGCAGGGTCCGAGGTATT-3ʹ

### Gait analysis

Gait analysis performed to assess the claudication of the affected limb induced by OA. Briefly, the animals were placed in a 100 × 10 cm open gait arena and allowed to freely walk from one side to the other in the absence of an external stimulus or food enticement. Outcome measures were obtained by three independent examiners who were blinded to the experimental conditions.

### Fluorescence molecular tomography (FMT)

The FMT 4000 In Vivo Imaging System from PerkinElmer, Inc. was utilized to monitor the levels of MMPs and cathepsins in the rat knee joints at 2 months after surgery. The rats received a single dose of MMPSense 645 FAST Fluorescent Imaging Agent (20 µl, 0.8 nmol; PerkinElmer, Inc.) and ProSense 750 FAST (20 µl, 0.8 nmol; PerkinElmer) via tail vein injection at 24 h before imaging. MMPSense is able to detect MMP-2, MMP-3, MMP-7, MMP-9, MMP-12 and MMP-13, while ProSense can detect cathepsins B, L, S, K, V and D, as well as plasmin. The concentrations of MMPs and PRO probes in the rat knee joints were calculated by using the region of the interest method, and the data are expressed as the means ± standard deviation (SD; n = 5 rat per group).

### Nanoindentation measurements

Nanoindentation is widely used to detect the mechanical properties of tissues or materials on a small scale. In this study, the microelasticity of human cartilage samples was determined using a novel nanoindenter. We used a probe with a 5.17 N/m spring constant and a 25 µm spherical indentation tip. During indentation, the spherical tip is in contact with the sample surface; loading indentation and loading time data are recorded. Probe displacement and probe displacement velocity were set to 10 µm and 18 µm/s, respectively. Young's modulus is derived from load-indentation curves using the Hertz model. The model was applied to the loading dataset corresponding to 80% of the maximum loading points; five unique points were measured per sample.

### Statistical analysis

Data are presented as mean ± standard deviation (SD). Histograms for RT-qPCR were generated using GraphPad Prism Software (version 5.0; Graph Prism Software, Inc.). SPSS version 19.0 was used to perform the analysis of variances. Pairwise comparisons were then performed using the least significant difference (LSD) multiple comparisons test.

### Ethics approval and consent to participate

This study is reported in accordance with ARRIVE guidelines (https://arriveguidelines.org). The animal experimental protocol strictly followed the “Guidelines for the Care and Use of Laboratory Animals” issued by the China Animal Research Committee and approved by the Animal Care and Use Committee (IACUC) of the Second Hospital of Shanxi Medical University (Taiyuan, China) (SYDL2019010).

## Results

### miR-1 downregulates Ihh expression and catabolism in rat chondrocytes

**Figure 1:**
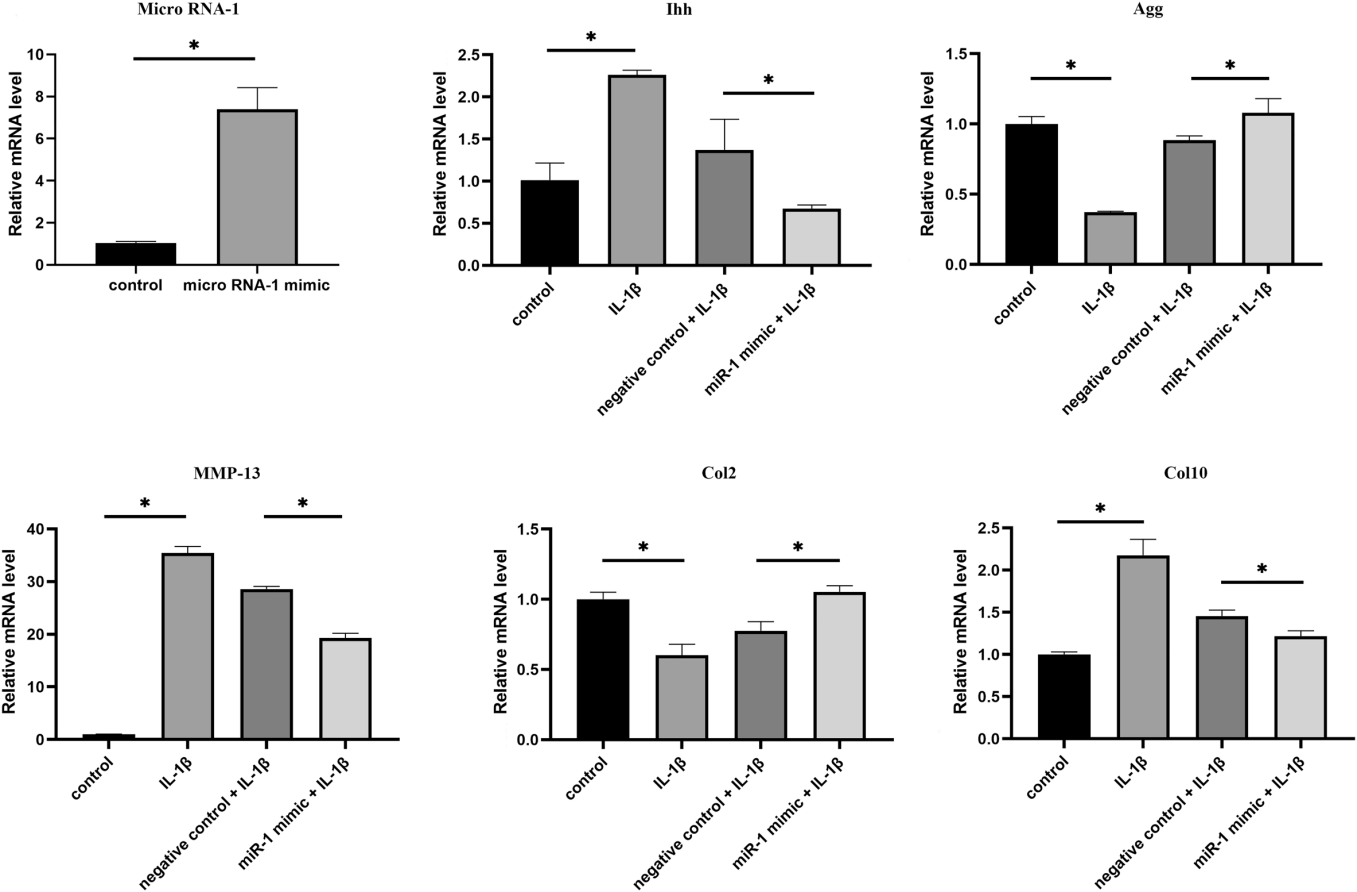
Effects of MiR-1 mimics on in vitro catabolism of IL-1itro catabolismiR-1 mimics on. *P < 0.05.

Our in vitro RT-qPCR showed that chondrocytes treated with miR-1 mimics demonstrated significantly increased miR-1 expression (Fig. 1). Additionally, IL-1β stimulation in cell culture increased Ihh, MMP-13, and Col10 expression, but reduced Col2 expression (Fig. 1). While MiR-1 mimic-treated cartilage cells showed reduced Ihh, MMP-13, and IL-1β-induced Col10 levels and increased Col2 and aggrecan (Agg) levels (Fig. 1).

### miR-1 attenuates the progression of osteoarthritis

**Figure 2:**
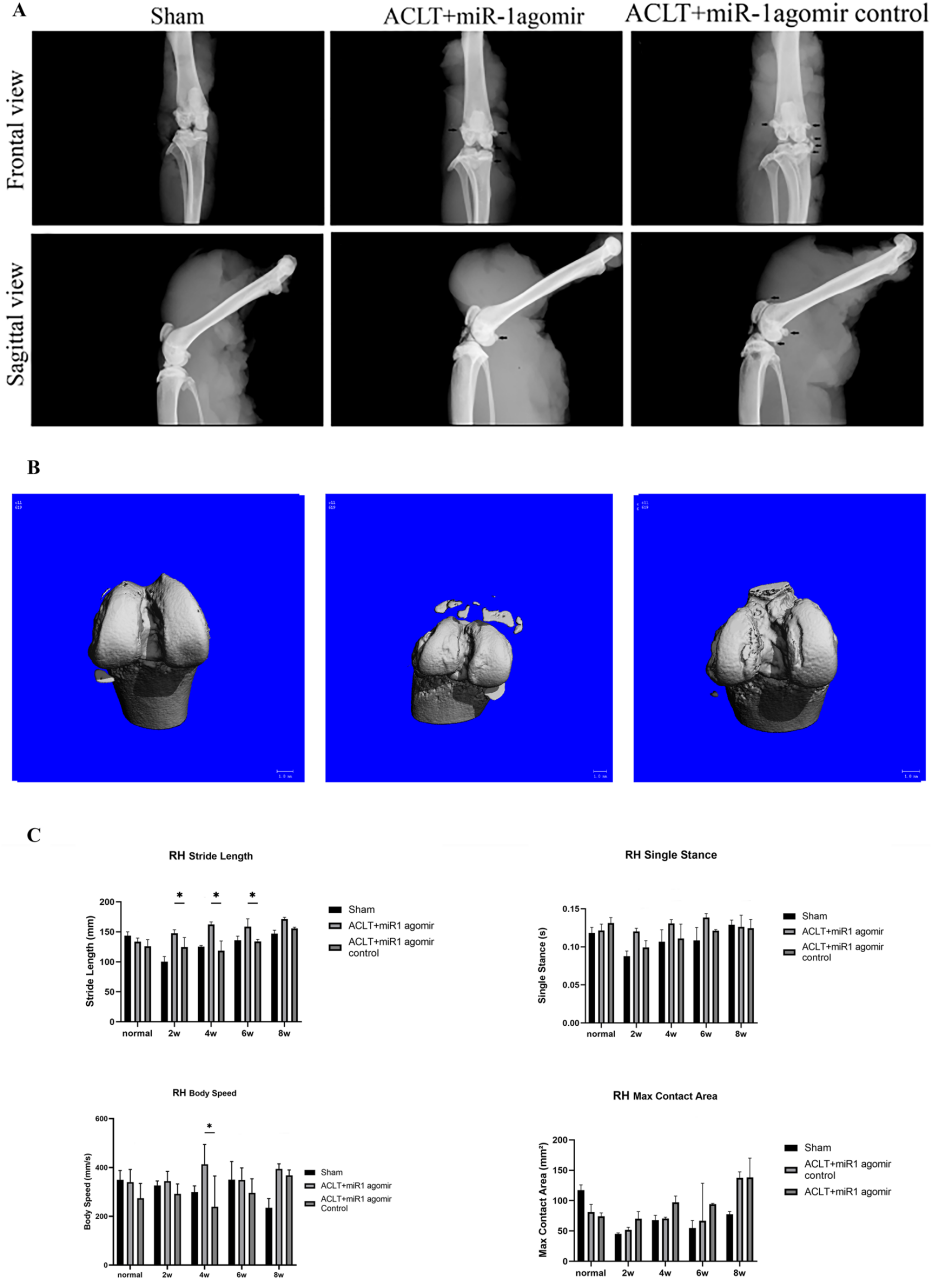
(**A**) Comparison of osteophyte formation in the frontal and sagittal X-rays eight weeks after ACLT. (**B**) Comparison of CT detection of osteophyte formation eight weeks after ACLT. (**C**) Comparison of gait analysis after ACLT. *P < 0.05.

Compared with the sham group, both the ACLT + miR-1 agomir and ACLT + miR-1 agomir control groups showed an increased number of osteophytes in the patella, tibial plateau, and tibial intercondylar eminence eight weeks after ACLT. However, the number of osteophytes in the ACLT + miR-1 agomir group was less than that in the ACLT + miR-1 agomir control group. Moreover, the degree of subchondral bone deformation in the ACLT + miR-1 agomir group was lower than that in the ACLT + miR-1 agomir control group. This shows that miR-1 agomir can attenuate OA progression (Fig. 2A, B). Gait analysis showed that compared with the control group, knee injection of miR-1 agomir could increase the stride length of rats two, four, and six weeks after ACLT and the movement speed of rats four weeks after surgery (Fig. 2C).

### miR-1 attenuates the severity of cartilage degeneration in OA

**Figure 3:**
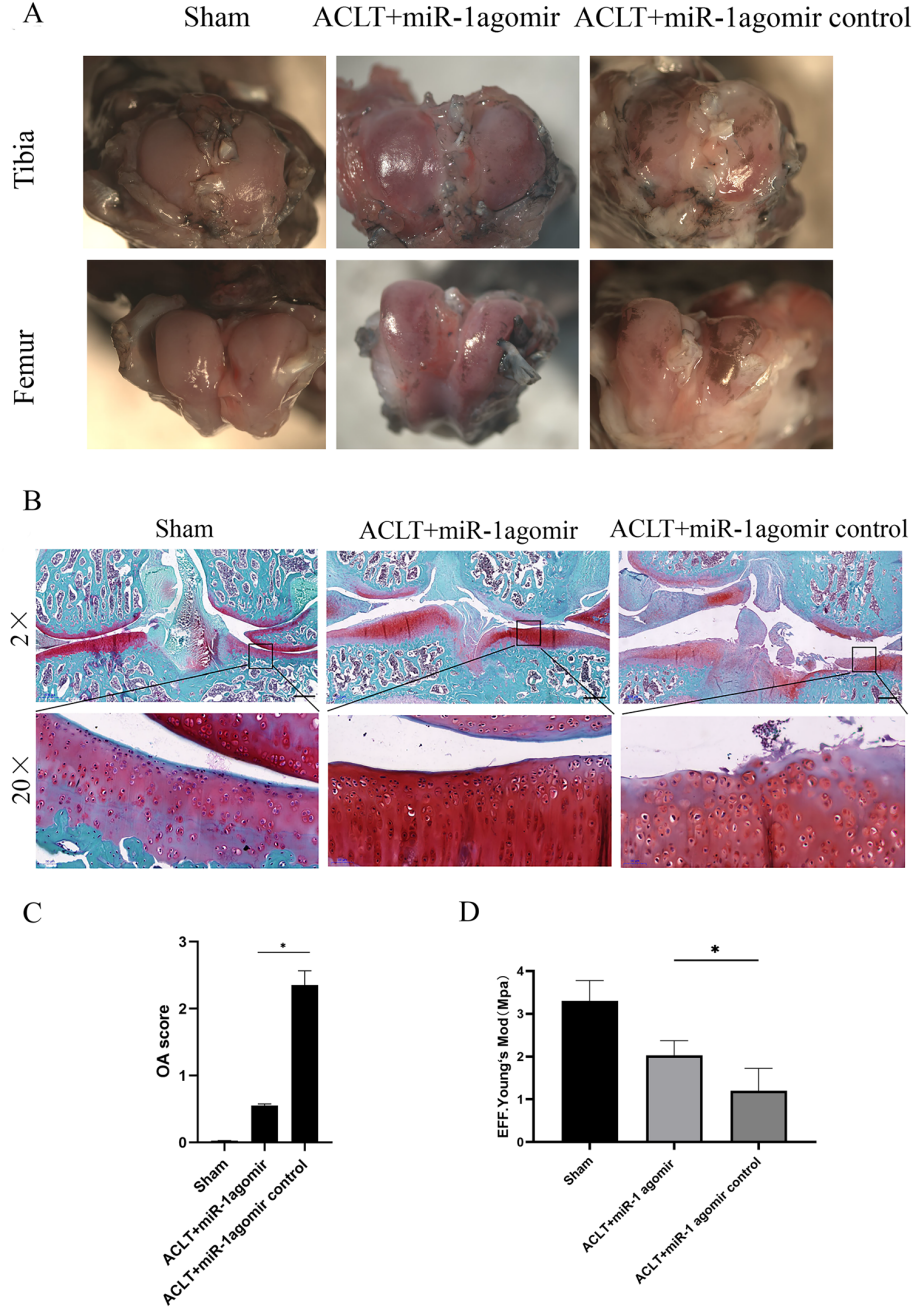
Histological comparison of experimental groups eight weeks after ACLT. (**A**) India ink staining points out damage with an arrow.The india ink figure Quantity is poor (**B**) Safranin O staining, bars: 500 µm. (**C**) Comparison of OA scores eight weeks after ACLT. (**D**) Comparison of nanoindentation eight weeks after ACLT. *P < 0.05.

Comparing among the three groups with each other, the sham operation group had the lowest Indian ink and the ACLT + miR-1 agomir control group had the highest Indian ink. This implies that arthritis in the sham operation group was the mildest, and arthritis in the ACLT + miR-1 agomir control group was the most severe. These results indicate that miR-1 agomir attenuates the severity of cartilage degeneration in OA (Fig. 3A).

Compared with the sham group, the articular cartilage surface of the ACLT + miR-1 agomir group was not flat enough. However, the ACLT + miR-1 agomir group was darker in red and had a flatter joint surface than the ACLT + miR-1 agomir control group. We also showed that miR-1 agomir attenuated the progression of OA (Fig. 3B). The OA score of the ACLT + miR-1 agomir control group (2.31 ± 0.56) was significantly higher than that of the ACLT + miR-1 agomir (0.53 ± 0.08; *P* < 0.05). Rats in the sham-operated group had the lowest OA scores (Fig. 3C). Nanoindentation showed that compared with the ACLT + miR-1 agomir control group, the injection of miR-1 agomir into the knee joint could upregulate Young's modulus of the tibial plateau cartilage in rats after ACLT and improve the elasticity of the cartilage (Fig. 3D).

### miR-1 promotes anabolism and inhibits catabolism of cartilage

**Figure 4:**
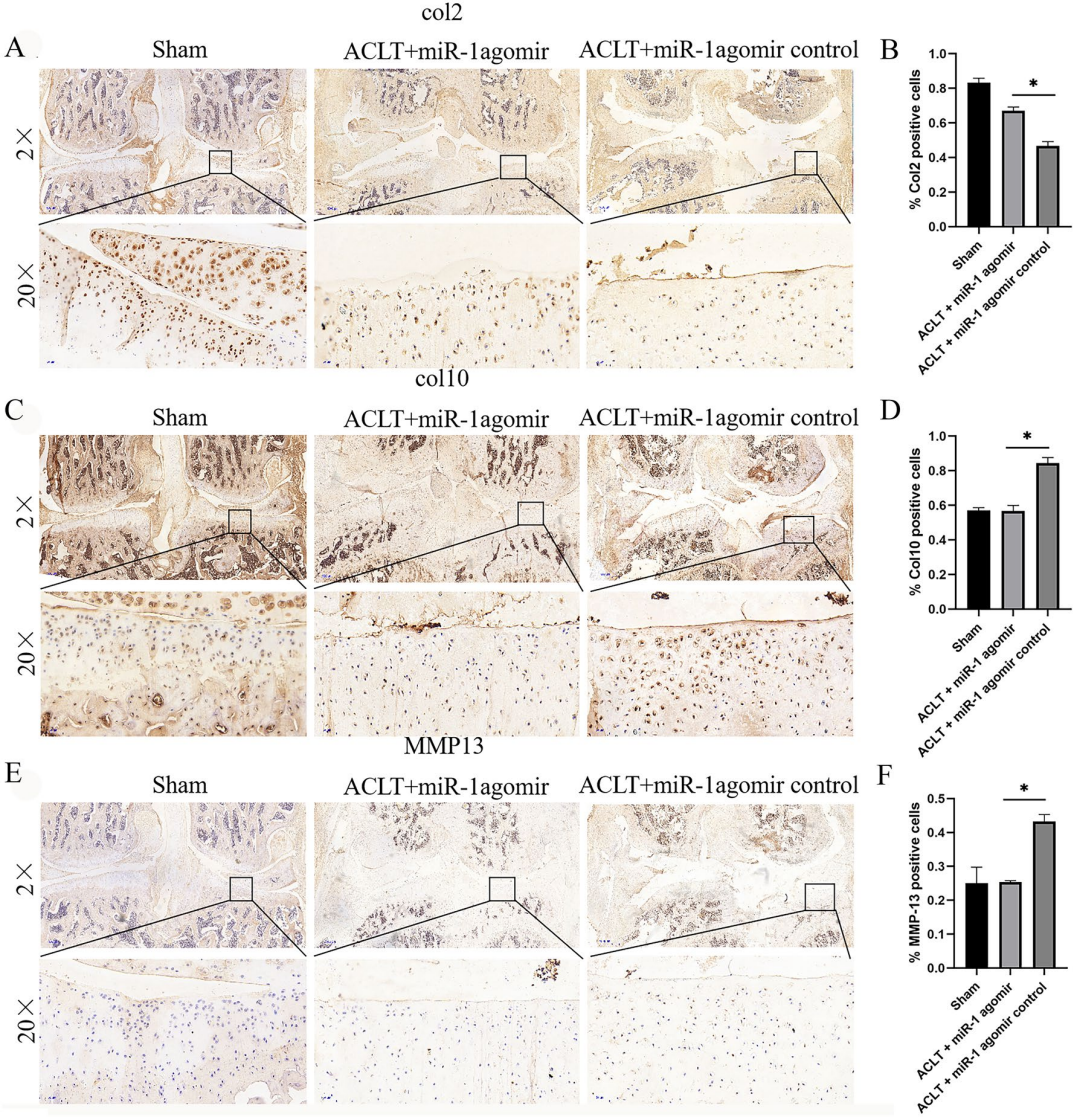
Different expression of proteins in an OA rat model eight weeks after surgery. (**A**) IHC staining for Col2 expression. (**B**) Quantification of Col2 positive cells based on the staining results. (**C**) IHC staining for Col10 expression. (**D**) Quantification of Col10 positive cells based on the staining results. (**E**) IHC staining for MMP-13 expression. (**F**) Quantification of MMP-13 positive cells based on the staining results. ACLT, anterior cruciate ligament transection; MMP, matrix metalloproteinase; Col2, collagen type II; Col 10, collagen type X.

Col2 expression was the most common in the sham operation group, followed by the ACLT + miR-1 agomir group, and the lowest in the ACLT + miR-1 agomir control group (Fig. 4A, B). This shows that miR-1 can promote cartilage anabolism. In contrast, both Col10 collagen and MMP-13 were the most abundant in the ACLT + miR-1 agomir control group, followed by the ACLT + miR-1 agomir group, and the least abundant in the sham operation group (Fig. 4C–F). This result indicates that miR-1 can inhibit cartilage catabolism.

### RT-qPCR indicates that miR-1 downregulates the expression of Ihh and its’ downstream genes

**Figure 5:**
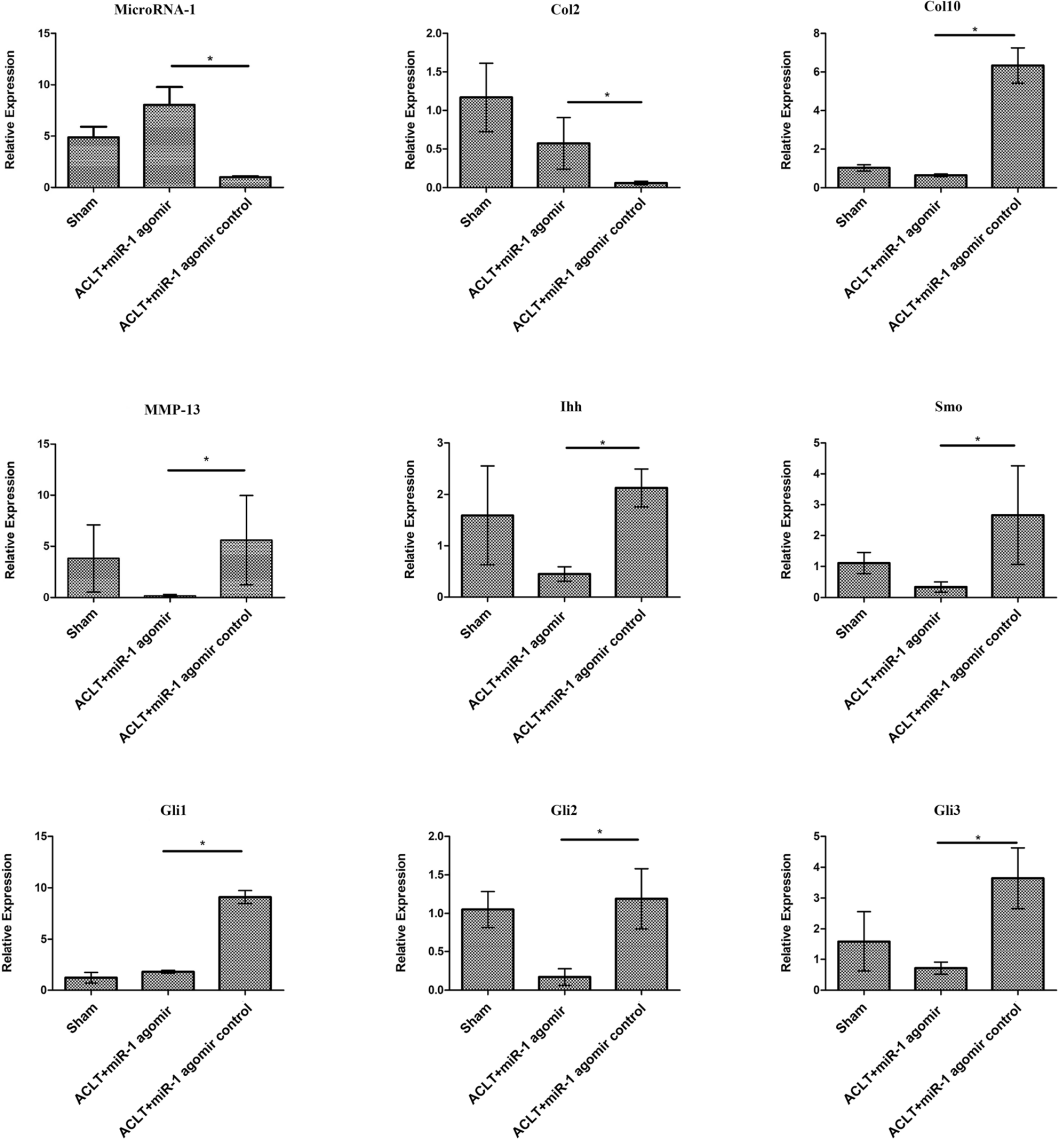
Effect of miR-1 overexpression on the inhibition of disease progression in an OA rat model eight weeks after surgery.

RT-qPCR results showed that the expression of miR-1 and Col2 in the ACLT + miR-1agomir group was higher than the ACLT + miR-1agomir control group. In addition, the expressions of Col10, MMP-13, Ihh, Smo, Gli1, Gli2, Gli3 in the ACLT + miR-1agomir group were lower than the ACLT + miR-1agomir control group (Fig. 5).

### miR-1 decreases MMP and prosense levels

**Figure 6:**
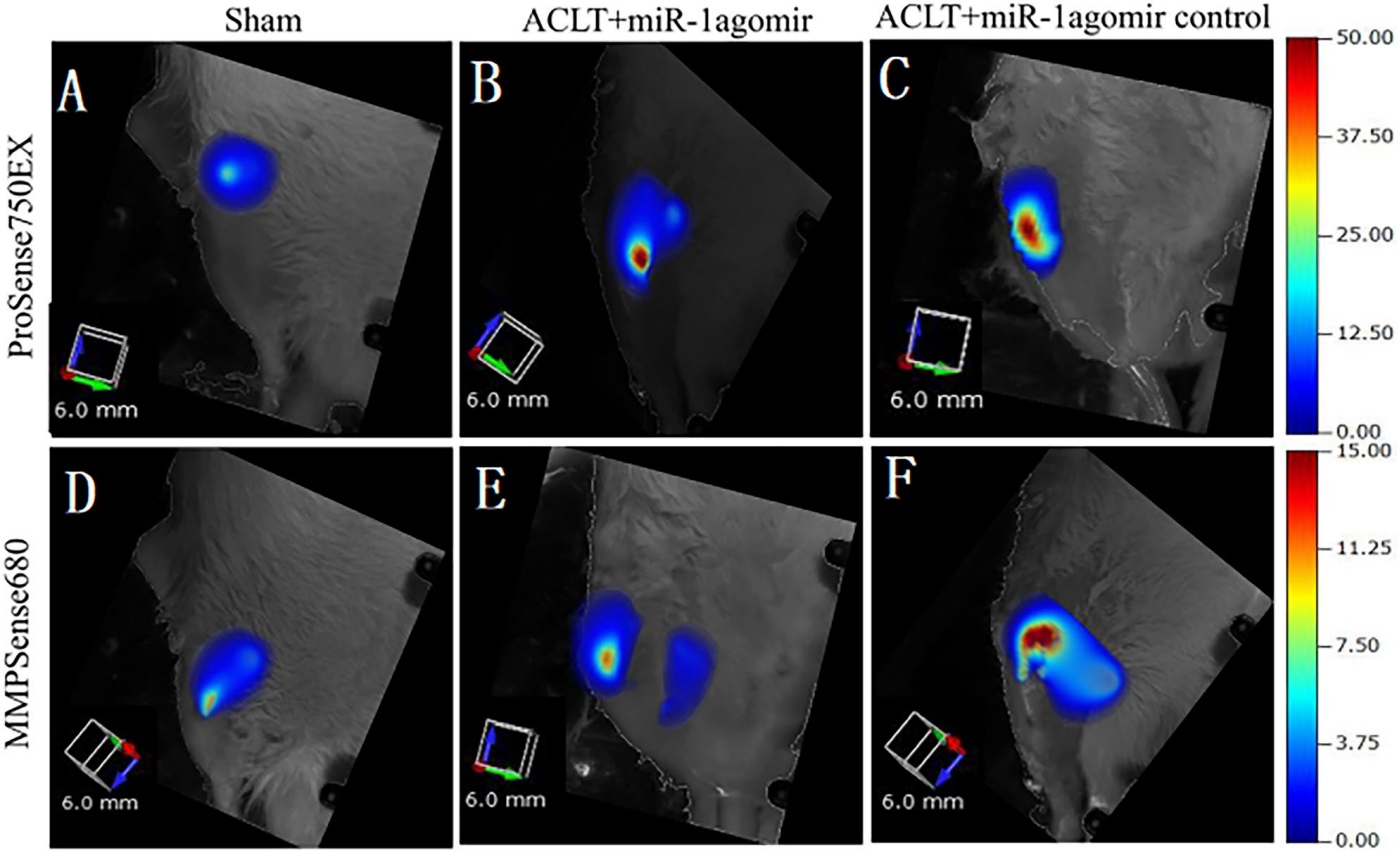
In vivo FMT measurement of total MMPs and cathepsins in an OA rat model eight weeks after surgery.

FMT results showed that MMP and prosense levels were significantly lower in the sham operation and ACLT + miR-1 agomir groups than in the ACLT + miR-1 agomir control group (Fig. 6). These results were consistent with the results of histology, IHC, and RT-qPCR.

## Discussion

OA is a common clinical joint disease often accompanied by articular cartilage degeneration and damage, which in turn causes changes in joint morphology, chronic joint pain, and even disability^22^. Many studies have shown that cartilage degradation in OA is accompanied by abnormal differentiation of articular chondrocytes. This process is similar to chondrocyte differentiation during cartilage osteogenesis^23^. During the development of OA, normal chondrocytes undergo pre-hypertrophy, hypertrophy, terminal differentiation, and mineralization. Specific gene expression changes significantly in each differentiation stage: healthy chondrocytes mainly secrete type II collagen and agglomerates; prehypertrophic chondrocytes mainly secrete Ihh; and hypertrophic chondrocytes specifically secrete type X collagen and matrix MMP-13. MMP-13 can further decompose the cartilage matrix, accelerate cartilage calcification, and promote OA development^24^.

In recent years, the role of Ihh in OA has attracted considerable attention. Ihh is a secreted protein that mainly plays a role in endochondral ossification, chondrocyte differentiation, proliferation, and maturation^25^. Ihh plays a complex role in the process of cartilage osteogenesis; the abnormal differentiation of chondrocytes in OA is similar to the differentiation process of chondrocytes in the process of cartilage osteogenesis^26^. Moreover, Ihh is expressed in prehypertrophic chondrocytes^27^, suggesting that Ihh upregulation promotes the hypertrophic phenotype and induces the expression of the typical hypertrophic markers Col10 and MMP-13. Therefore, the upregulation of Ihh signaling promotes OA progression. Our previous study showed that the disruption of the Ihh signaling pathway in vivo attenuated OA progression in a transgenic mouse, using an Ihhfl/fl model of surgically induced OA^11^. Thus, upregulation of the Ihh pathway plays an important role in OA progression, whereas inhibition of the Ihh pathway attenuates cartilage degradation.

In this study, we constructed an in vitro model of OA by stimulating rat chondrocytes with IL-1β. This resulted in decreased expression of Col2 and increased expression of Col10, Runx-2, and MMP-13. We first used this OA in vitro model to investigate the protective effects of miR-1 mimics on rat chondrocytes. Our results showed that miR-1 mimics improved the effects of IL-1β in rat chondrocytes and downregulated Ihh expression. We further established an osteoarthritis model using eight-week-old SD rats and injected miR-1 small nucleic acid drug into the joint cavity one week later. We found that eight weeks after surgery, miR-1 small nucleic acid drug reduced the tibial plateau and uplifted the tibia in OA. The number of superfluous formations and joint planes was flatter and Smoother. India ink staining indicated various severity levels of arthritis. The larger the area covered by India ink, the more severe is the arthritis. After the injection of miR-1 small nucleic acid drugs into the joint cavity, the stained area of India ink was smaller and arthritis was reduced. The results of safranin O staining showed that after the injection of miR-1 small nucleic acid drugs into the joint cavity, it had a darker coloration and flatter joint surface. The results of immunohistochemistry showed that the injection of miR-1 small nucleic acid drugs into the joint cavity reduced the consumption of Col2 and production of Col10 collagen and MMP-13, which promoted bone anabolism and reduced bone catabolism. FMT results further showed that after the injection of miR-1 small nucleic acid drugs into the joint cavity, the levels of MMP and prosense were significantly reduced, which is associated with the progression of OA. RT-qPCR results showed that after injection of miR-1 small nucleic acid drugs into the joint cavity, the expression levels of Ihh and its downstream related genes, Smo, Gli1, Gli2, and Gli3 decreased, the expression levels of MMP-13 and Col10 decreased, and the expression levels of Col2 increased. The results of this study indicate that intra-articular injection of miR-1 small nucleic acid drugs reduces OA progression by inhibiting Ihh.

Recently, an increasing number of studies have been conducted on the treatment of OA by intra-articular injection of miRNA, and adverse side effects are limited, which may provide new ideas for the future treatment of OA^28,29^. Yang et al. suggested that the inhibitory effect of miR-1 on Wnt/β-catenin signaling in chondrocytes might attenuate the expression of genes that regulate the activity of catabolic enzymes. This finding may be useful for further studies on the molecular targets for OA treatment^30^. The research results of Chen et al. showed that the overexpression of miR-1 promoted the expression of collagen (Col)-II and aggrecan, which are related to matrix synthesis. However, all these studies were conducted using subangular tubes for intra-articular injection of drugs^31^. Shoji and Kawanishi et al. reported that intra-articular injection of miRNA-210 and atelocollagen effectively promoted the healing of partially torn ACL and damaged meniscus^32,33^, Peng et al. demonstrated that intra-articular injection of lentiviral targeting drug SFs containing miRNA140 precursor molecules could improve autoimmune arthritis^34^. In many previous studies, intra-articular gene delivery relied on viral or non-viral delivery systems such as non-collagen, lentivirus, and adenoviral vectors^34,35^. Although they have shown good safety in recent years, they may also exhibit biological toxicity and even lethal biological toxicity^35^. Direct modification of known drugs is a widely used therapeutic strategy. This method can extend the residence time of drugs and effectively reduce biological toxicity^27^. However, there are few studies on drugs that can modify miRNAs in the articular cavity. Si et al. injected miR-140 into the joint cavity of OA rats, which reduced the progression of osteoarthritis in rats without complications, indicating that microRNA agomir intra-articular injection may be a method for the treatment of OA^9^.

This study had several limitations. Firstly, an anterior cruciate ligament cut operation was used to induce OA. Although this is a classic method for establishing an OA model^36^, it is still different from the clinical development process of OA. The etiology of osteoarthritis is multifactorial because the disease is often caused by aging and trauma in the clinic^37^. Secondly, in this study, we mainly focused on the effect of miRNA-1 on chondrocytes and cartilage but did not study its effect on other cells (fibroblasts, osteoblasts/osteoclasts, and meniscus cells) or tissues (synovial cells, subchondral bone, and meniscus). Thirdly, we only injected a single dose of miRNA-1 agomir into the joint to treat early OA. However, different doses and treatment courses and different treatment intervals (one month or longer) affect different stages of OA and need further research. Finally, this study utilized three experimental groups in the animal model, lacking both an OA model group and a Sham + miR-1 agomir group, thus precluding observation of the impact of miR-1 agomir on normal cartilage. Subsequent investigations are planned to address this limitation and expand upon the findings of this study.

In summary, our results show that intra-articular injection of miRNA-1 small nucleic acid drugs can alleviate OA progression by inhibiting the Ihh pathway in rats. The intra-articular application of miRNAs may be a potential method for the treatment of OA, and more comprehensive studies are necessary to test the clinical potential of miRNAs in the treatment of OA.

## Data Availability

All data generated or analyzed during this study are included in this published article.
